# Assessment of the Minimum Clinically Important Difference in Symptoms and Functions of Patients With Acute Schizophrenia: A *Post hoc* Analysis of an Open-Label, Single-Arm Multicenter Study

**DOI:** 10.3389/fpsyt.2021.653916

**Published:** 2021-05-03

**Authors:** Tianmei Si, Chuan Shi, Ling Sun, Yilong Zhang, Lili Zhang

**Affiliations:** ^1^Peking University Sixth Hospital (Institute of Mental Health), Beijing, China; ^2^NHC Key Laboratory of Mental Health (Peking University) & National Clinical Research Center for Mental Disorders (Peking University Sixth Hospital), Beijing, China; ^3^Tianjin Anding Hospital, Tianjin, China; ^4^Xian Janssen Pharmaceuticals, Beijing, China

**Keywords:** schizophrenia, minimum clinically important difference, positive and negative syndrome scale, personal and social performance, clinical global impression-severity scale

## Abstract

The purpose of this study was to evaluate the application of the minimum clinically important difference (MCID) concept to clinical results in Chinese patients with acutely exacerbated schizophrenia. The original study was an 8-week, open-label, single-arm, multicenter study of flexible doses of paliperidone-extended release (pali-ER) in Chinese patients with acutely exacerbated schizophrenia. This is a *post hoc* analysis to determine the MCID value of PANSS, PSP and evaluate the responsiveness of each outcome measurements in the acute phase of schizophrenia. The responsiveness of the four measurements (PANSS, PANSS reduction rate, PSP, CGI-S) was analyzed. Four hundred ninety nine patients completed the 8-week follow-up and were finally used for this *post hoc* analysis. The MCID calculated by different approaches varied from 14.02 to 31.50 for PANSS, 15.14 to 42.79% for PANSS reduction rate, and 7.62 to 13.13% for PSP. In addition, the improvement of the CGI-S owned the highest responsiveness of the four outcome measurements. The threshold value of MCID for schizophrenia patients was determined by choice of the assessment method to an extent. In addition, the CGI-S score appeared to be the most valid and responsive measure of effectiveness for the acute phase of schizophrenia when take the treatment satisfaction of patients as anchor.

## Introduction

Schizophrenia is a psychiatric disorder with a lifetime prevalence of 0.3–0.66%. It can lead to a considerable psychosocial dysfunction (1) and can influence the quality of life of patients significantly, resulting in the need for assistance in meeting basic living needs (1, 2). The Positive and Negative Syndrome Scale (PANSS) and the Personal and Social Performance (PSP) assessment are reliable tools for assessing the symptoms and functional outcomes in the acute and stable stages of schizophrenia (3). PANSS is the most widely used standardized scale for assessing symptom severity in schizophrenia (4). It has been used as an outcome measure in a multitude of treatment efficacy studies and is increasingly used in clinical practice. The PSP scale was designed to measure and distinguish between specific domains of functioning and includes specific criteria for rating the severity of dysfunction, in addition to incorporating psychopathologic aspects of behavior. PANSS and PSP are based on summary rating scores and lack a gold standard to interpret results. Clinicians must rely on the experience with individual patients and populations to interpret PANSS scores and the clinical significance of various degrees of change (5).

A majority of studies have attempted to quantify the efficacy of the therapeutic intervention and report changes in group means before and after treatment. However, group means cannot be readily used in clinical practice to interpret changes on an individual basis, while statistical significance differentiates generalizable differences from those that exist by chance. A large sample can reveal statistically significant differences that are not clinically meaningful (6). Therefore, the concept of “minimum clinically important difference” (MCID) has been presented as an alternative tool to quantify clinically significant patient improvements due to a therapeutic intervention. MCID is defined as the smallest change that is meaningful to patients and is considered the threshold needed to achieve treatment efficacy (7). An MCID value in a given study that exceeds a threshold value indicates that a clinically significant change is achieved, which may validate a “decision to treat” (8).

The perception of psychosocial functioning may vary among different cultures. The MCID value of PANSS and PSP for Chinese patients with schizophrenia still remains unknown. It was, therefore, of interest to conduct a China-specific study for investigating the MCID in this population.

An 8-week, open-label, single-arm, multicenter study showed favorable efficacy, safety, and tolerability profiles of flexible doses (3–12 mg/day) of paliperidone-extended release (pali-ER) in Chinese patients with acutely exacerbated schizophrenia (9). A *post hoc* analysis of this study was conducted to determine the MCID value of PANSS, PSP and CGI-S, and evaluate the responsiveness of each outcome measurements in the acute phase of schizophrenia.

## Methods

### Patient Sample

This primary study was an 8-week, open-label, single-arm, multicenter study to evaluate the efficacy, safety, and tolerability of flexible doses of pali-ER (3–12 mg/day) tablets in patients with acutely exacerbated schizophrenia. The study was conducted at 20 sites in China.

Patients (age ≥18 years) of either sex, diagnosed with acute schizophrenia (based on the *Diagnostic and Statistical Manual of Mental Disorders, 4th Edition*, criteria) and a PANSS total score of ≥70 at baseline, were enrolled in the study. Patients diagnosed with substance dependence (current or within the previous 6 months), a history of tardive dyskinesia or neuroleptic malignant syndrome, or a significant risk of suicide or violent behavior were excluded from the study.

Patients were hospitalized within the first 7 days after study initiation. Follow-up visits were scheduled in weeks 2, 4, and 8. This *post hoc* analysis included individuals who completed an 8-week follow-up.

### Outcome Assessment

#### PANSS

Symptoms of schizophrenia were measured according to the total score on PANSS. In 1987, Kay et al. published the PANSS and the PANSS manual to address the limitations of existing instruments for schizophrenia research (4). The choice of these 30 items was made with the objective of obtaining content validity for both the positive and the negative symptoms of schizophrenia, as well as the associated general psychopathology (10). The PANSS is a reliable and valid instrument that has served the scientific research community well for decades. As Hermes (11) pointed out, the PANSS is a set of 30 questions scored from 1 to 7, and hence the PANSS total score has a minimum value of 30 points, making the calculation of percent change in the PANSS score problematic. In this study, both the PANSS total score change and the PANSS reduction rate were calculated.

#### PSP

The PSP was designed to measure and distinguish between specific domains of functioning and included specific criteria for rating the severity of dysfunction, in addition to incorporating psychopathologic aspects of behavior (12). The PSP consisted of items across four domains: socially useful activities, personal and social relationships, self-care, and disturbing and aggressive behavior. The scores on the PSP scale ranged between 1 and 100, with higher scores indicating better personal and social functioning.

### Clinical Global Impression—Severity Scale

The Clinical Global Impression—Severity scale (CGI-S) is a clinically intuitive scale widely used to assess illness severity in patients with schizophrenia (13). It provides a single score using a 7-point scale: 1 = “not at all ill”; 2 = “very mildly ill”; 3 = “mildly ill”; 4 = “moderately ill”; 5 = “markedly ill”; 6 = “severely ill”; and 7 = “extremely severely ill.” Higher scores indicated greater severity of psychotic symptoms.

### Anchor

The treatment satisfaction of patients at an 8-week follow-up was used as the anchor for the derivation of anchor-based MCID calculations. The treatment satisfaction of patients assessed how a patient felt at the time of the questionnaire completion compared with 8 weeks previously. Satisfaction was converted into a numeric scale of 1–5 (1 = “extremely satisfied;” 2 = “satisfied;” 3 = “neither satisfied nor dissatisfied;” 4 = “dissatisfied;” 5 = “not at all satisfied”).

### Approaches for Calculating MCID

No “gold standard” methodology exists for estimating the value of MCID. A majority of methods fall into the following two categories: distribution-based methods and anchor-based methods. One distribution-based and two previously reported anchor-based approaches were chosen: (1) “change difference,” the difference in the average change score between responders and nonresponders (14, 15); (2) “minimum detectable change” (MDC), the smallest value that is greater than the measurement error within a 95% confidence interval (CI) (16, 17); and (3) “receiver operating characteristic (ROC) curve,” a sensitivity- and specificity-based approach for calculating the MCID.

### Responsiveness of the MCID Value of Each Outcome Measurement

The consistency of each outcome measurement with the anchor ROC curve was used to evaluate the consistency of four outcome measurements (PANSS, PANSS reduction rate, PSP, and CGI-S) with the anchor. The accuracy of the ROC curve was evaluated using the calculated area under the curve (AUC). AUC in the range of 0.90–1.00 was considered excellent, 0.80–0.90 was considered good, 0.60–0.80 was considered fair, and 0.50–0.60 was considered to indicate failure (18). Thus, to evaluate the responsiveness of the MCID value of each outcome assessment, the AUC of the ROC curve and Spearman's correlation coefficient (*r*) were used to determine the relationships between responses to the anchor. The correlation coefficient *r* was a number between −1 and +1; numbers 0.10–0.29 referred to a weak, 0.30–0.49 to a moderate, and 0.50–1.0 to a large correlation (19).

### Statistical Analysis

All statistical analyses were performed using SPSS (version 19.0, Inc., USA). The baseline and 8-week scores were compared using the Mann–Whitney *U* test. Unless stated otherwise, the hypothesis test was two-sided with 0.05 significance; a *P*-value ≤ 0.05 was considered significant.

## Results

### Patient Characteristics

The description of the study sample is shown in Table 1. Of the 608 enrolled patients, 499 (82.1%) completed the 8-week follow-up and were finally used for this *post hoc* analysis. The mean age of the patients at baseline was 32.23 ± 11.1 years. Of the patients, 50.1% (250/499) were female, and 49.9 % (249/499) were male. All patients received 6 mg pali-ER at the beginning and on average 7.83 ± 2.19 mg during the 8-week study, with a daily dose range of 3–12 mg.

**Table 1:** Demographic characteristics of participants.

**Variable**	**Mean or *n* (% or SD)**
Age (year)	32.23 (SD = 11.51)
Sex (male)	249 (49.9%)
Duration of illness (year)	7.58 (SD = 8.75)
**CGI-S**	
Moderately ill	47 (9.4%)
Markedly ill	227 (45.5%)
Severely ill	218 (43.7%)
Among the most extremely ill patients	7 (1.4%)

### MCID Threshold Values for the Outcome Measurements

All outcome measurements showed a significant improvement after pali-ER treatment with an 8-week follow-up (Table 2). The comparison of different anchor- and distribution-based approaches yielded a wide range of MCID threshold values for each outcome measure (Table 3). These values varied from 14.02 to 31.50 for PANSS, 15.14 to 42.79% for PANSS reduction rate, and 7.62 to 13.13% for PSP.

**Table 2:** Outcomes (PANSS, PSP, and PANSS reduction rate) at baseline and 8-week after treatment.

**Outcome score**	**Baseline**	**8 weeks after treatment, mean (SD)**	***P*-value**
PANSS	89.40 (13.53)	49.84 (15.23)	<0.01
PSP	44.00 (12.82)	69.02 (14.61)	<0.01
PANSS reduction rate	–	43.66% (16.88%)	–

**Table 3:** MCID threshold values for PANSS, PANSS reduction rate, and PSP.

**MCID calculation method**	**Outcome measures**		
	**PANSS**	**PSP**	**PANSS reduction rate (%)**
MDC	18.77	7.62	23.39
Change difference	14.02	13.13	15.14
ROC curve derived	31.50	12.50	42.79

### Consistency of Each Outcome Measurement With the Anchor

**Figure 1:**
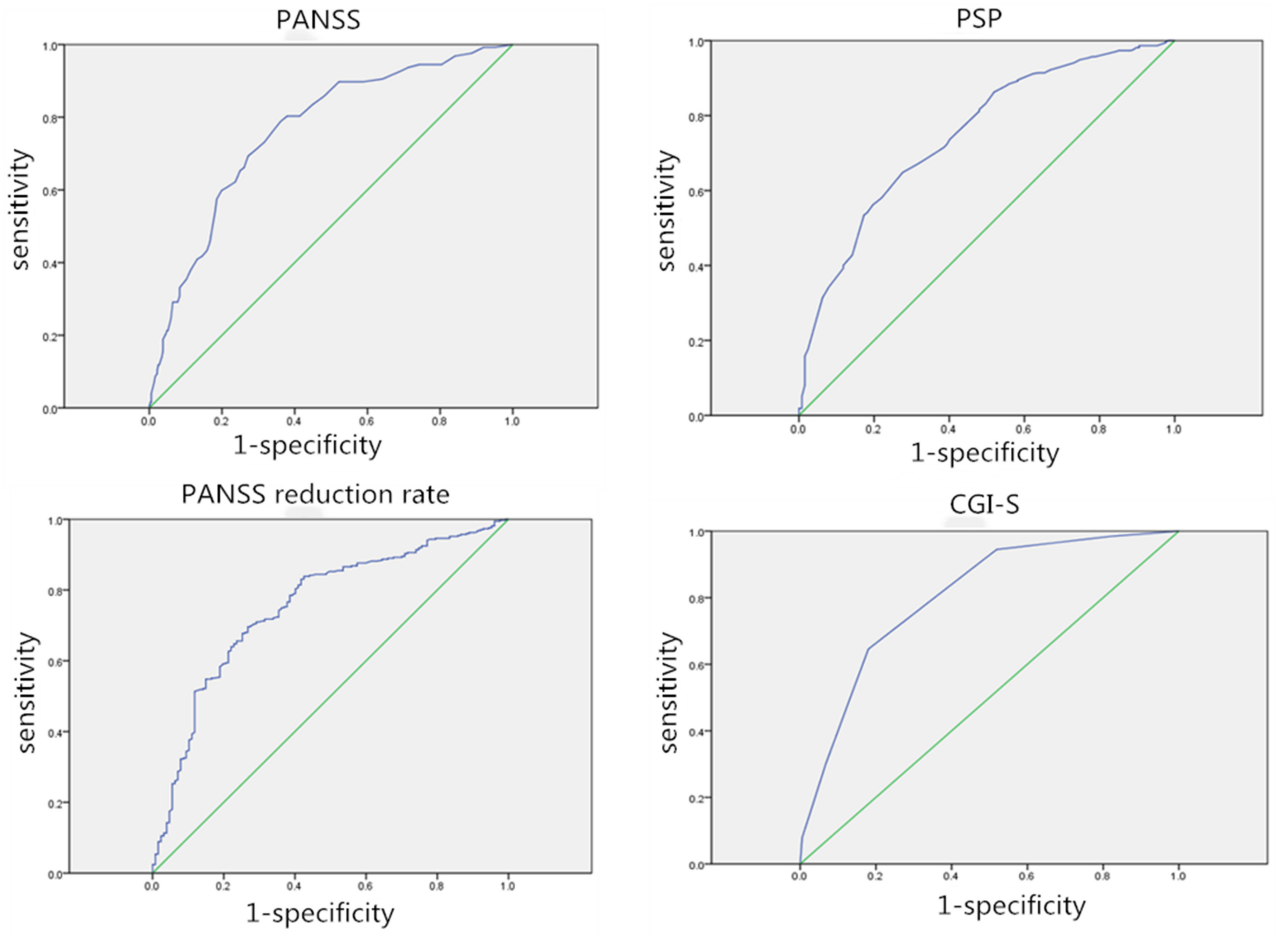
Receiver operating characteristic (ROC) curves for each outcome assessment. AUC, Area under the curve; ROC curve, receiver operating characteristic curve.

The ROC curve was used to compare four outcome measures (PANSS, PANSS reduction rate, PSP, and CGI-S) assessed in this study to determine which outcome measure was the most valid and responsive measure of therapeutic effectiveness in patients with acutely exacerbated schizophrenia. Besides, the association between the responses to the anchor and the change in outcome measurements was also calculated. The AUC varied from 0.750 to 0.804, indicating that the ROC curve exhibited suitable accuracy in discriminating between responders and non-responders (Figure 1). The AUC for the PANSS, PANSS reduction rate, PSP, and CGI-S was 0.762, 0.755, 0.750, and 0.804, respectively. And the cut-offs for PANSS, PANSS reduction rate, and PSP were 31.5, 42.79 and 12.5, respectively. The CGI-S appeared to be the most accurate discriminator of meaningful effectiveness (AUC of 0.804) and the most responsive measure to postoperative improvement. Correlations were found to be large for CGI-S (*r* = 0.559), moderate for PANSS (*r* = 0.392) and PANSS reduction rate (*r* = −0.484), and weak for PSP (*r* = −0.295) within the treatment satisfaction.

## Discussion

This study estimated the value of MCID for the PANSS and PSP in patients with acutely exacerbated schizophrenia, which is a widely used measure of symptoms and functions in schizophrenia. The current study, a *post hoc* analysis of data from a study conducted on Chinese patients, revealed significant differences in PANSS and PSP changes from baseline to 8 weeks using flexible doses (3–12 mg/day) of pali-ER. The MCID range for patients in the acute phase of schizophrenia was 14.02–31.50 for PANSS, 15.14–42.79% for PANSS reduction rate, and 7.62–13.13 for PSP.

### Choice of the MCID Calculation Approach

Many methods are used to calculate the MCID value, and no consensus exists regarding the best method. The anchor-based and distribution-based methods all have arbitrary components (8).

The distribution-based methods generally use the statistical characteristics of the sample, such as the standard deviation to separate “signal” from “noise” (5, 11). The MDC represents the smallest change in an outcome measure score that exceeds the measurement error. For meaningful effectiveness, the MCID value must be at least equal to, or greater than, the MDC (20, 21). MDC was consistently greater than the measurement error (allowing for the reliable interpretation of true change in treatment effectiveness). However, distribution-based calculations do not incorporate the patient perspective. Also, the SD may vary in patient populations, which means that this method does not provide information about the clinically important size of the change and should ideally be linked to a clinical measure of the MCID.

The anchor-based measures require the patient to report a perceived change in clinical status. The limitations of the anchor-based methods include the availability of multiple potential anchors to use. One tool presumed to have clinical meaning is the Clinical Global Impression Scale, which is frequently used as an external standard in the anchor-based methods of estimating the MCID (2). However, a limitation of this method is that CGI scores are based on the subjective perceptions of clinicians and their ability to recall the clinical status of the patient at baseline and are influenced by their previous or concurrent experiences. For this reason, CGI-P (a patient-rated measure) and obtaining employment (an objective external standard of recovery) were used as anchors to estimate the MCID value for patients with schizophrenia.

Perhaps it is not possible to find a fixed value that can represent the MCID, but still, an approximation above the measurement noise is possible. In this study, an anchor-based approach was chosen to produce an externally referenced MCID established by independent questioning, and a distribution-based method was incorporated to produce a reasonable MCID range.

### Analysis of the Population and Comparison With Other Studies

Several previous studies on a heterogeneous population have attempted to find out the MCID value of PANSS and PSP for patients with schizophrenia. The estimated range of MCID provided by the present analysis (14.02–31.50 for PANSS, 15.14–42.79% for PANSS reduction rate, and 7.62–13.13 for PSP) was substantially greater than that previously reported.

Cramer (22) described an MCID value of 21% for PANSS reduction. The analysis enrolled 423 patients with treatment-resistant schizophrenia at multiple Veterans Affairs sites with a 12-month follow-up, and a 5-level clinician-rated global clinical change scale was used as an anchor. Hermes et al. (11) reported an MCID value of 15.3 points or 34.0% from baseline for the PANSS by linking PANSS with CGI-S scores and 11.2 points (24.6%) for CGI-P. However, Leddy-Stacy (2) addressed a markedly lower MCID value (4.25–8.30) for PANSS using obtaining employment as an anchor. Interestingly, both Hermes and Meaghan used the data from the Clinical Antipsychotic Trials of Intervention Effectiveness trial, which is one of the largest and longest schizophrenia trials conducted to date, comparing the effectiveness of multiple antipsychotic treatments using broad inclusion criteria in a variety of treatment settings. Nasrallah (12) reported a 7-point improvement in the PSP by analyzing pooled data from two phase 3, 52-week, multicenter clinical trials.

As indicated earlier, the estimated value of MCID in the present study was larger than that in previous studies, which might be explained as follows: (1) A patient-reported anchor was used in the present study; different anchors might have caused different MCID values even for the same population. (2) Several authors suggested that the MCID value for the PANSS varied depending on the severity and chronicity of symptoms (11, 23). Different from previous studies, only patients diagnosed with acute schizophrenia were enrolled in the present study. Based on the aforementioned two reasons, the result might be more relevant for patients with acutely exacerbated schizophrenia.

### Consistency of Each Outcome Measurement With the Anchor

The ROC curve and correlations between responses to the anchor were calculated to evaluate which assessment was the most valid and responsive measure of the therapeutic effectiveness. The ROC curve was used to identify the threshold for outcome measures while maintaining the greatest sensitivity and specificity possible and to quantify personal bias. The CGI-S owned the highest AUC, and the correlation coefficient between the CGI-S and the anchor reached 0.559, which was larger than the correlation coefficient between the anchor and the PANSS, PANSS reduction rate, and PSP. The CGI-S appeared to be the most accurate indicator of meaningful effectiveness and most responsive to patient satisfaction. Leddy-Stacy (2) suggested that clinicians likely used overall evaluations of improvement, comprising judgment of improvement in multiple domains. Symptom control in patients with schizophrenia is considered the mainstay approach for achieving physical and cognitive improvements, although it may not always result in better functional outcomes. Improvement in the overall quality of life and social functioning of patients is still a challenge (24). Thus, it is important to note that no comprehensive evaluation method is available for schizophrenia yet. Although CGI-S is a simple evaluation method, it takes improvement in multiple domains into account, making it more accurate than PANSS and PSP.

### Strengths

The strengths of this study were the large number of patients (*n* = 499), 20 sites in one country, and the high completion rate of 82.1%.

### Limitations

The present study had limitations that might have affected the optimal analysis. First, as the data were from a phase-3 clinical trial, participation in the trial might have introduced some potential selection biases. In addition, the population under study was restricted to patients with a single diagnosis and using one specific antipsychotic. As a result, it may be difficult to assess whether some of the variations in MCID thresholds demonstrated in this study were actually due to statistical artifacts. Still, all four scales were assessed in the same condition, which might mitigate the biases to some extent. Second, the patient group was highly homogeneous, limiting the generalizability of the results. Third, the anchor used in this study was subjective and not comprehensive, and the lack of an objective external anchor might have limited the ability to identify the most representative MCID calculation method. Finally, it would have been desirable to investigate whether the MCID values differed according to high or low baseline scores. Unfortunately, that was not possible because the patient group was highly homogeneous.

### Conclusions

The threshold value of MCID for schizophrenia patients was determined by choice of the assessment method to a great extent. The MCID value was 14.02–31.50 for PANSS, 15.14%−42.79% for PANSS reduction rate, and 7.62%−13.13% for PSP. In addition, the CGI-S score appeared to be the most valid and responsive measure of effectiveness for the acute phase of schizophrenia when take the treatment satisfaction of patients as anchor.

## Data Availability Statement

The original contributions presented in the study are included in the article/supplementary material, further inquiries can be directed to the corresponding author/s.

## Ethics Statement

The study protocol and amendments were reviewed by the Shanghai Mental Health Center Institutional Review Board. The study was conducted in compliance with the Declaration of Helsinki consistent with Good Clinical Practices and applicable regulatory requirements. Written informed consent was obtained from all patients before enrollment.

## Author Contributions

TS and CS designed the protocol and oversaw data collection. LZ and LS conceptualized the hypotheses, conducted statistical analyses, and interpreted the data. YZ and TS wrote the first draft of the manuscript. LS and YZ helped to conceptualize the study, interpret data, and contributed to the manuscript. All authors reviewed the results and approved the final version of the manuscript.

## Conflict of Interest

YZ and LZ were employees of Xian Janssen Pharmaceuticals at the time during which the work was undertaken. This work is a post-hoc analysis of a Xian Janssen sponsored phase 3 study R0776477-SCH-3034. The remaining authors declare that the research was conducted in the absence of any other commercial or financial relationships that could be construed as a potential conflict of interest.
